# N-3 Polyunsaturated Fatty Acids and Inflammation in Obesity: Local Effect and Systemic Benefit

**DOI:** 10.1155/2015/581469

**Published:** 2015-08-03

**Authors:** Yue Wang, Feiruo Huang

**Affiliations:** Department of Animal Nutrition and Feed Science, College of Animal Science and Technology, Huazhong Agricultural University, Wuhan 430070, China

## Abstract

Overwhelming consensus emerges among countless evidences that obesity is characterized by a chronic low-grade inflammation in the adipose tissue (AT), which subsequently develops into a systemic inflammatory state contributing to obesity-associated diseases. N-3 Polyunsaturated fatty acids (n-3 PUFA), known as important modulators participating in inflammatory process, turn out to be an effective mitigating strategy dealing with local and systemic inflammation observed in obesity. Some of the effects of n-3 PUFA are brought about by regulation of gene expression through interacting with nuclear receptors and transcription factors; other effects are elicited by modulation of the amount and type of mediator derived from PUFAs. The metabolic effects of n-3 PUFA mainly result from their interactions with several organ systems, not limited to AT. Notably, the attenuation of inflammation in hard-hit AT, in turn, contributes to reducing circulating concentrations of proinflammatory cytokines and detrimental metabolic derivatives, which is beneficial for the function of other involved organs. The present review highlights a bridging mechanism between n-3 PUFA-mediated inflammation relief in AT and systemic benefits.

## 1. Introduction

There are mounting evidences demonstrating that obesity and associated disorders pose a daunting threat on global public health problem, given their morbidity and mortality. Obesity is associated with a chronic low-grade inflammatory response characterized by abnormal cytokine production, increased acute phase reactants, and activation of inflammatory signaling pathways [1, 2]. It is specifically the excessive accumulation of body fat in AT that initiates local inflammation and later systemic response [3]. AT is more like an important endocrine and immune organ, secreting a considerable variety of adipokines, including inflammatory mediators [3, 4]. Increased circulating concentration of these cytokines is triggered by obesity and proven associated with pathogenesis of metabolic syndrome [1, 5]. That partly explains why obese individual is vulnerable to serious complications, ranging from diabetes to cardiovascular disease. AT inflammation is never considered as a mere local reaction. Instead, locally inflamed AT exerts profound influences on other organs and systems.

The anti-inflammatory properties of n-3 PUFA are not novel concepts, and the benefits of dietary supplementation with n-3 PUFA are well documented in several inflammatory and autoimmune diseases, including obesity [6, 7]. Under the condition of obesity, catabasis of inflammation manifestation mediated by n-3 PUFA is never restricted to AT. In parallel, it indeed benefits the improvement of other metabolic sites. According to our observation, even during normal physiological processes, n-3 PUFA suppresses the expression of proinflammatory cytokines and decreases the circulatory TNF-*α* concentration [8]. It is conceivable that the anti-inflammatory potential of n-3 PUFA might be stretched under the inflammatory status. However, it seems reasonable to suppose that n-3 PUFA may mediate some of its beneficial effects on whole-body homeostasis by resetting the function of AT. Since the peroxisome proliferator-activated receptor-gamma (PPAR*γ*) functions as an effector of adipocyte-derived T helper 2 (Th2) cytokines [9] whose activation is required for the circulating monocytes differentiation into M2 macrophages and anti-inflammatory gene transcription, as discussed later in this review, typically, PPAR*γ* might function as communicator integrating inflammation relief in AT into systemic benefits. The emphasis of present review is upon benefits from n-3 PUFA supplementation in models of chronic inflammatory conditions companied with obesity. On a global level, the potential adipocentric beneficial effects are expended into systemic effects. Given its desirable anti-inflammatory effect, dietary manipulation with n-3 PUFA may therefore offer a logical strategy for preventing or treating obesity and obesity-induced complications.

## 2. Adipose Tissue Inflammation in Obesity

Interestingly, inflammation emerging in the context of obesity seems to be triggered and to reside predominantly, in the AT [10], although other organs actively involved in metabolism also inevitably suffer from a chronic low-grade inflammation during the development of the diseases. More than a notable characteristic of obesity, AT inflammation is considered as causative factors connecting obesity with its metabolic complications [3, 11–13].

In addition to matrix of extracellular proteins, adipocytes are surrounded by a wide variety of cells, including endothelium, fibroblasts, preadipocytes, and immune cells [14]. In contrast to previous understanding, adipocytes are increasingly recognized as an integrator of various physiological pathways rather than a passive energy storage depot with a droplet which is completely out of the proportion [15]. This recognition begins with the observation that adipocyte is a significant source of endogenous tumor necrosis factor*α* (TNF-*α*) at which secretion is substantially stimulated by obesity [10, 16]. Adipocytes are equipped with the ability to secrete a large number of adipokines (interleukin-6 (IL-6), interleukin-1 (IL-1), chemokine monocyte chemotactic protein, leptin, adiponectin, resistin, etc.) [15]. Following adipocytes, resident immune cells constitute the second largest AT cellular component [3]. Obesity-induced infiltration of immune cells into AT leads to increased synthesis and subsequent secretion of proinflammatory factors into circulation [17].

Obesity is featured by progressive infiltration of immune cells into AT. Adipocytes are studied with increasing intensity, while some pursuit concentrates on the possibility that the cellular source of these inflammatory changes derives not only from adipocytes. Actually, since the majority of cytokines produced in obese AT are adipose tissue macrophage (ATM) derived, it is speculated that recruitment and proinflammatory activation of ATM are required for the development of obesity-associated complication [18, 19]. Histologically, there is evidence of significant infiltration of macrophages into white adipose tissue (WAT) of obese mice, which well supports earlier microarray analysis demonstrating that gene product expressed in macrophage is markedly increased in obese (*ob/ob*) AT [20–22] and unequivocally explains previous discoveries that macrophage accumulation is positively correlated with body mass index (BMI) and adipocyte size [14, 21]. With the deterioration of obesity, macrophages recruit in the AT, sometimes contributing as much as half of the cellularity [21]. The state of chronic low-grade inflammation in WAT is powerfully augmented through the infiltration of macrophages [23]. Instead, fat reduction leads to a dramatic decrease in number and modified distribution of macrophages together with decreased expression of inflammatory markers [22]. Not all ATM are programmed for proinflammation, since even, for lean mice, moderate quantity of ATM is present in the context of low or undetectable inflammatory signals [21]. Diet-induced obesity leads to a shift in the activation state of ATM from an M2-polarized state in lean animals to a proinflammatory M1 polarization state [24]. The mechanism responsible for this phenotypic switch of macrophage polarization remains unclear. It is speculated that dietary n-3 PUFA servers as the monitor switch, which will be discussed in following context. It is worthwhile to mention that macrophage, overwhelmingly localized to necrotic adipocytes, is the predominant or significant source of proinflammatory adipokines in WAT [25]. Adipocyte death is one of the putative mechanisms explaining the initiation of macrophage infiltration into adipose [26].

In addition to macrophages infiltration, adipocyte hypertrophy and hyperplasia are followed by enhanced angiogenesis and extracellular matrix (ECM) overproduction. The protein composition and dynamics of ECM are critical for physiologic role of the AT [27]. Additionally, an emerging view is that adipocytes hypertrophy sets obstacle for sufficient oxygen supply to the cells, which creates a state of hypoxia followed by apoptosis of some cellular components [28, 29], once physical limit to adipocyte growth hardly copes with increased volume. Virtually, if disproportionate accumulation of ECM does not allow an adequate expansion of adipocytes, adipocytes are more susceptible to necrosis [30]. Presumably, as a process pathologically accelerated in obesity, unhealthy AT remodeling, not AT expansion, may be the root of the attraction of macrophages and final inflammation, at least necrosis adipocytes mainly caused by nutrition and oxygen deprivation are unignorable stimulus that drives immune cells infiltration as discussed before. More important, in spite of an enlarged fat mass, well controlled healthy AT expansions do not exhibit pathological changes and metabolic disorders [26].

Another marked feature of obesity is the dramatic upregulation of Inflammation and macrophage-specific genes in AT [1]. Earlier microarray performed in AT from wild-type and* ob/ob* mice [20] and subsequent research concerning the treatment with PPAR*γ* ligand in Zucker diabetic fatty (ZDF) rats [31] both disclose the notable regulation of inflammatory genes in AT during obesity development. In addition, extensive transcriptional profiling studies using multiple tissues taken from mice with genetic or diet-induced obesity indicate the majority of gene regulated in obesity comprises macrophage and inflammatory genes [1]. Similar conclusion is observed in the WAT of mice suffering from varying degrees of obesity [21].

Although macrophage invasion into visceral adipose tissue (VAT) is regarded as a dominant driving force leading to AT inflammation, there has also been a resurgence of interest into a unique population of VAT-resident regulatory T (T_reg_) cells characterized by the expression of forkhead box P3 (Foxp3). T_reg_ cells-mediated suppression turns out to be an effective defense against aberrant or excessive immune responses [32]. T_reg_ cells are abundant in the VAT of normal but not obese individuals, suggesting that they are engaged in control of the inflammatory state of AT [33]. What is more, they have a different T cell receptor repertoire compared with T_reg_ cells residing in other tissues, which also indicates their novelty. Interestingly, VAT T_reg_ cells reduce strikingly and specifically in insulin-resistant models of obesity. Conversely, their expansion improves insulin sensitivity [33]. Loss-of-function and gain-of-function experiments further confirm their influence on the surrounding adipocytes, inflammatory state of AT, and insulin sensitivity, coupled with differentially synthesized cytokines [33]. It is likely that the shortage of VAT-resident T_reg_ cells offers the answer why obesity-associated inflammation can escape the powerful armamentarium of cells and molecular ready for curbing a runaway immune response.

**Figure 1:**
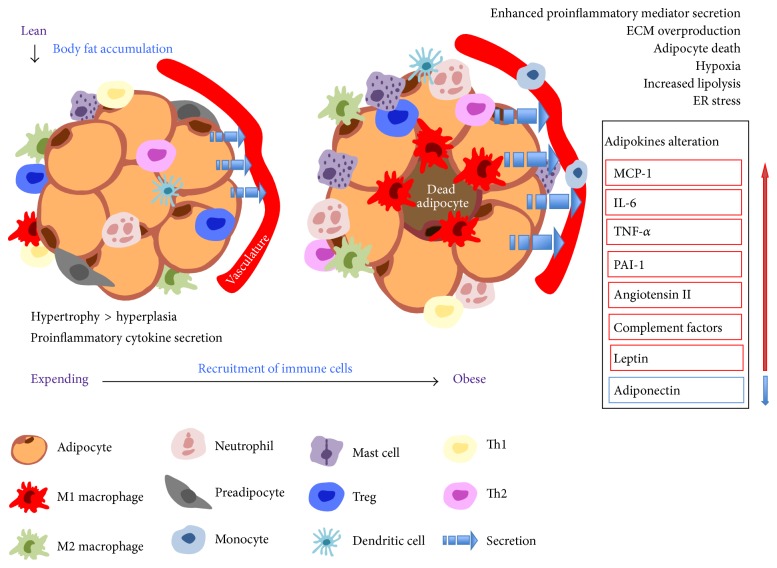
Alteration occurring in adipose tissue when it suffers from excessive TAG accumulation. In the face of chronic overnutrition, excessive adipose tissue expansion is initiated and dominated by adipocyte hypertrophy, whereas adipocyte number becomes fixed with obese individuals achieving a higher plateau. Activated adipocytes trigger production of proinflammatory mediators, together with resident immune cells, exerting functions in an endocrine and paracrine fashion. Infiltration of immune cells is secondary consequence followed by adipocyte activation. Mature adipocytes promote diapedesis of monocytes through microvascular endothelial cells, facilitating monocyte-derived macrophages accumulation. Progressive recruitment of immune cells underpins inflammation in AT by enhancing proinflammatory mediator secretion. Additionally, obese state pathologically accelerates AT remodeling featured by ECM overproduction, necrotic adipocyte, and hypoxia, along with dysregulation in fatty acid fluxes. Macrophages are predominantly localized around dead adipocytes. Sensibly, lean individuals exhibit higher ratios of M2:M1 macrophages, Th1:Th2 T cells. With the development of obesity, major shift in the above cell ratios favors a modest inflammation status (originality is inspired by previous review written by Evan D. Rosen and Bruce M. Spiegelman. Based on former illustration focusing on recruitment of immune cells, more detail and latest information are injected into the figure).

Exposed to obesity-susceptive environment, complex events take place in AT in a defined order with a number of critical cell types participating in this process, as shown in Figure 1.

## 3. Systemic Consequences in Response to Local Inflammation

Elevated TNF-*α* expression in AT from rodent models of obesity was first proposed in 1993 [16]. Subsequently, the novel function of TNF-*α* is verified in different rodent obesity models as well as in obese humans [10, 34–36]. The absence of TNF-*α* results in significantly improved insulin sensitivity in both diet-induced and* ob/ob *model of obesity [37]. In sharp contrast to elevated TNF-*α* expression in AT, circulating TNF-*α* concentrations in obesity are found unchanged or disproportionately increased [16, 38, 39]. Demonstration of elevated expression of transmembrane forms of TNF-*α* in obesity well confirms the spatial restriction of TNF-*α*, suggesting the action of this cytokine is restricted to AT because of TNF-*α* retention on the cell surface [36]. Attempts to reverse insulin resistance with an injection of anti-TNF binding proteins finally fail [40, 41], indirectly supporting the speculation that TNF-*α* functions locally at AT via a paracrine or autocrine fashion. However, it would be premature to conclude that AT inflammation is an isolated system.

TNF-*α* is able to amplify inflammatory response via activating other cytokine networks and proinflammatory pathways [42]. Apart from TNF-*α*, there is a significant graded increase of proinflammatory cytokines, such as IL-6 as well as acute phase markers, which work in a coordinated manner to impact whole-body hemostasis. More direct and detailed discussions concerning the linkage between these cytokines and inflammation associated diseases are reported before [42]. Studies concerning the origins of the accumulated triacylglycerol (TAG) in the liver during the development of nonalcohol fatty liver disease clarify that the primary contributor to hepatic TAG is serum nonesterified fatty acid (NEFA) pool, most of which derived from AT fatty acid flux [43]. Enhanced release of fatty acid from hypertrophic fat cells results in lipotoxicity caused by accumulation of lipid in nonadipose tissues, contributing to systemic insulin resistance [44]. However, a recent discovery, contrary to previous demonstration, not only challenges former opinion by confirming downregulated rates of NEFA delivery from AT, but also demonstrates that the implicit reduction in AT fatty acid uptake goes beyond the downregulation of systemic NEFA release from AT in obesity [45]. No matter what mechanism dominates the excess fat deposition in nonadipose tissue, accretive fatty acid release originated from AT or impaired AT storage of ingested fat, the dysfunction, and destruction necessarily associated with ectopic fat depots cannot be too strongly emphasized. Ectopic fat depots with predominantly systemic effects include VAT, intrahepatic fat, and intramuscular fat, whereas pericardial (or the related epicardial or pericoronary fat), renal sinus fat, myocardial steatosis, and perivascular fat are postulated to have potential local effects [46].

By analogy to proinflammatory mediators and free fatty acid (FFA), several adipokines, especially leptin and adiponectin protecting peripheral tissues against the lipotoxic damage by promoting oxidation of fatty acids and sensitizing insulin actions, are also an integral part of AT-derived factors which bridge the physiology of AT and function of non-AT tissues [44]. Unfortunately, obesity is always accompanied with increased leptin and decreased adiponectin in serum [47], negatively associated with desirable metabolic parameters.

A direct relationship between ATM with other metabolic or nonmetabolic disarrangements suggests that ATM plays integrated role that goes beyond local AT inflammation [48], with emphasizing on the effects of polarization state of ATM on systemic inflammation and insulin action [49, 50]. The mechanism underlying this process remains deeper investigation. According to achieved evidences, it is clear that vastly increased production of proinflammatory mediators derived from M1 macrophages results in their entry into the circulation to cause dysfunction of actively metabolized tissue.

In conclusion, the label of endocrine organ reflects the systemic effects of AT. Excessive accumulation of surplus body fat lies at the core of all these problems and initiates the release of a number of proinflammatory cytokines from adipocytes as well as tissue-resident macrophages, followed by a rapid recruitment of monocytes from circulation to the AT. Infiltrating cells deteriorate the state of inflammation by representing an additional source of proinflammatory cytokines, leading to disruption of normal homeostatic control of metabolism locally and systemically via endocrine or paracrine effects. In addition to dysregulation of secretory functions, impairment of storage function results in excessive release of free fatty acids and ectopic deposition in nonadipocyte cells, which dramatically worsens the situation [51, 52].

## 4. N-3 PUFA-Mediated Prevention and Reversal of Metabolic Syndrome

The structural feature, metabolic interconversion, and food source of n-3 PUFA are well demonstrated in previous literatures [53]. Numerous researches, in vivo or in vitro, confirm the positive effects of n-3 PUFA supplement on lipid and glucose metabolism, such as lower triacylglycerol concentration and higher high density lipoprotein (HDL) cholesterol levels in plasma, improved insulin sensitivity as well as reduced blood pressure [54, 55]. Animal dietary intervention trials demonstrate that n-3 PUFA limits development of obesity and reduces cellularity of AT in the context of the diet rich in fat, with improvement of lipid and glucose metabolism [56–58]. Importantly, n-3 PUFA administration alleviates high-fat diet (HFD)/obesity-induced insulin resistance, which is equal to or greater than the effects of clinically used insulin sensitizing drug [59]. Clinical studies in humans also report that n-3 PUFA contributes to a significant decrease of body fat and improves glucose metabolism and plasma lipid profile simultaneously [60–62]. The division of scientific opinions focuses on the issue that n-3 PUFA consumption significantly reduces food intake [63], since some clearly observe less weight again in animals fed fish oil-based diets, an effect cannot be explained by less lower caloric intake [64]. The discrepancy likely comes from the level of energy intake and experimental design, since the weight gain of control animals varies distinctly between different studies, and the conclusion is based on the comparison with control treatments different in fatty acid content. Notably, the metabolic and molecular effects of different high-fat diets with varying fatty acid compositions are systemically compared. Unlike other high-fat diets tested, n-3 PUFA dietary regimen appears to be the only treatment successful in fighting against high-fat induced metabolic deterioration associated with obesity [64].

As said before, inflammation is an inevitable feature in the development of obesity and contributes to obesity-related metabolic derangements. Conversely, ameliorating the low-grade inflammation companied with obesity by n-3 PUFA benefits obesity and its sequelae via modulating adipokines secretion and improving insulin sensitivity [44].

Actually, the ratio of dietary n-6 to n-3 PUFA, rather than the absolute amount of n-3 PUFA, is important in determining the development of inflammatory response [65, 66]. Linoleic acid and *α*-linolenic acid (ALA) are the precursors responsible for the synthesis of n-6 and n-3 series, respectively. Despite different metabolites, the enzymes involved in their metabolism turn out to be the same, which means excessive intake of linoleic acid leads to reduced synthesis of docosahexaenoic acid (DHA) and eicosapentaenoic acid (EPA) because of competition for relevant enzymes. Increased ingestion of n-6 PUFA also inhibits EPA incorporation into neutrophil membranes and reduces the inhibition of production of inflammatory mediators [67, 68]. But in vivo, n-3 PUFA prevents the arachidonic acid- (AA-) induced increase in proinflammatory eicosanoids in fat cells [69]. Given the inefficiency of transformation from ALA to n-3 long-chain polyunsaturated fatty acid (LC-PUFA) [70, 71], increasing dietary consumption of EPA and DHA helps to reach the maximum beneficial effects. Besides that, precise difference exists between DHA and EPA and their precursor ALA, because longer carbohydrate chains seem more potent to exert stronger effects [7]. In most cases, it is active metabolites derived from n-3 PUFA, rather than themselves, that exerts anti-inflammatory effects.

## 5. Anti-Inflammatory Effects of n-3 PUFA

Clinical investigations of dietary supplementation with n-3 PUFA indicate their beneficial impact on some certain diseases, especially those companied with inflammation [6]. Anti-inflammatory effects of n-3 PUFA are primarily demonstrated by two standpoints: inhibitory secretion of proinflammatory mediators [72, 73] and greatly reduced macrophage migration into AT [74]. On the other hand, as outlined in the later paragraphs, n-3 PUFA assists with body fat reduction by multiple mechanisms including prevention of adipocytes proliferation, increased fatty acid oxidation, and inhibited hepatic lipogenesis, which can be considered as an indirect way to exert its anti-inflammatory effects given the immune role played by AT. Last, incorporation of the n-3 PUFA into adipocyte membrane is also regarded as a metabolism for its favorable effect in AT. Alterations occurring in AT finally result in systemic benefits.

### 5.1. Production of Proinflammatory and Anti-Inflammatory Cytokines

Among these identified factors contributing to uncontrolled inflammation in obesity, special attention is given to bioactive lipid mediators derived from the cyclooxygenase and 5-lipoxygenase pathways, which convert the membrane-derived AA into potent proinflammatory eicosanoids (such as prostaglandin and leukotriene). Notably, active metabolites derived from PUFA indeed play indispensable roles in the development of inflammation [44]. In contrast to proinflammatory potential of eicosanoids derive from n-6 PUFA, eicosanoids derived from n-3 series are generally gifted in anti-inflammation [75]. A relatively small increase in n-3 LC-PUFA consumption significantly inhibits AA conversion to proinflammatory eicosanoids generated by the 5-lipoxygenase pathway of neutrophils, monocytes, and macrophages [76, 77]. Thus, increase in dietary n-3 PUFA can shift the balance of the produced eicosanoids to a less inflammatory mixture, resulting from competitive inhibition of proinflammatory cytokines conversion.

In parallel, an arsenal of new lipid mediators is isolated and identified, suggesting that these novel families of anti-inflammatory mediators contrast with the earlier n-3 PUFA-derived oxygenated products previously known as eicosanoids [78]. As we know, in most cases, inflammation is normally well regulated and followed by complete resolution that enables inflamed tissues to return to homeostasis due to the activation of negative feedback mechanisms, which means influx of specialized leukocytes induces mononuclear leukocytes recruitment to phagocytose apoptotic leukocytes and cells debris from inflamed site until the injurious stimulus is cleared and infiltrated leukocyte is removed [79]. Contrarily, chronic inflammation is featured by continual activation of the adaptive immune system that exacerbates the inflammatory response. Of special interests in this process are some previously ignored factors that signal the termination of inflammation. As endogenous local mediators, these bioactive substances derived from EPA and EHA during the resolution phase are termed protectins and resolvins, because specific members of these families are widely appreciated for their ability to stimulate and accelerate resolution [80, 81]. In addition to controlling the magnitude and duration of inflammation, resolvins and protectins also function as inflammation terminators facilitating removal of chemokines from the resolving milieu [78]. Emerging evidence demonstrates that resolvins and protectins exert a strict control towards the resolution process of AT inflammation and pave the way for monocyte migration and their differentiation into macrophages and even elicit macrophage polarization toward an M2-like phenotype [79, 82].

Besides the mediators derived from PUFA, it is confirmed that, for healthy volunteers, supplementation of n-3 PUFA suppresses the production of secreted proinflammatory mediators [83–85]. Indeed, monocytes pretreated with n-3 PUFA significantly decrease proinflammatory production after lipopolysaccharide (LPS) stimulation [86–88]. Aligning with the emerging phenotype, a conceivable reduction in the expression of a number of inflammatory markers is achieved, in partial analogy to the situation with anti-inflammatory drugs treatment [63].

### 5.2. Alteration Occurring in Adipose Tissue

In addition to dietary PUFA-originated metabolites and n-3 PUFA-mediated cytokines secretion, recent studies implicate n-3 PUFA influence on the polarization and recruitment of macrophages [52]. ATM in lean individuals is of an anti-inflammatory phenotype but capable of excessive proinflammatory mediator production [89]. Incorporation of n-3 PUFA in diet completely prevents macrophage infiltration induced by high-fat diet [90], which is consistent with another research revealing dramatic reduction in ATM content, along with transformation from M1 to M2 polarization state [59]. Similar macrophage phenotypic switch considered as a benefit of n-3 PUFA is observed in Kupffer cells [91].

Undeniably, adipocyte is the other target through which n-3 PUFA prevents AT inflammation. One possible mechanism lies in the prevention of AT expanding and proliferation of adipocytes by n-3 PUFA. Antiadipogenic effect of n-3 PUFA significantly prevents fat accumulation by reducing cellularity of AT, with a preferential inhibition in the epididymal fat [58]. Compatible with animal experiments, n-3 PUFA inhibits adipocyte differentiation, induces apoptosis in postconfluent preadipocytes, and promotes lipolysis [92]. Additionally, oxidation may be also involved in the profound effects of n-3 PUFA on prevention of enlarged adipocytes. The effect of n-3 PUFA in abdominal fat is associated with increased expression of genes engaged in mitochondrial biogenesis and oxidative metabolism, contributing to the shrinkage of adipocytes [93]. It is worthwhile to mention that dietary n-3 PUFA counteracts accretion of body fat without inducing mitochondrial uncoupling protein 1 (UCP1) in AT, suggesting that the antiobesity effect is independent of adaptive thermogenesis [94]. However, conflicting results give rise to the question whether the reductive effect on body fat accumulation exerted by n-3 PUFA is attributed to both limited hypertrophy and hyperplasia of adipocytes. Treatment with n-3 PUFA results in a significant decrease in the size of mature adipocytes and accumulation of smaller adipocytes [95], possibly along with increased total number of adipocytes. It cannot be excluded that the formation of small adipocytes results not only from shrinkage of existing mature adipocytes, but also from the proliferation of preadipocyte due to the activation of PPAR*γ* [96]. Taken together, it is reasonable to speculate that n-3 PUFA ameliorates adipocyte stress and normalizes adipocyte functions, especially secretory function.

**Figure 2:**
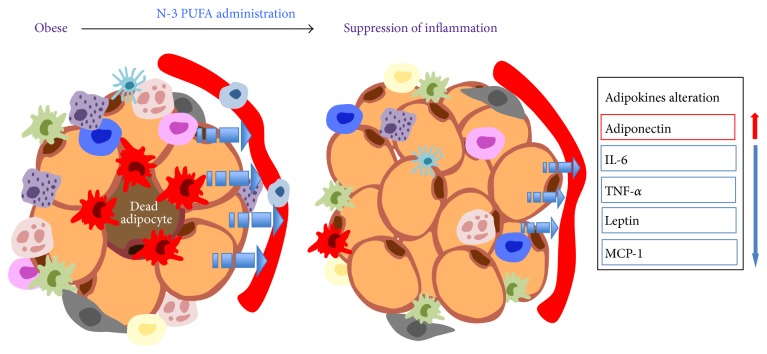
Mitigation of AT local inflammation by n-3 PUFA as a result from normalization of AT storage and secretory function. In the presence of n-3 PUFA, it is clear that the population of small adipocytes increases, possibly with unchanged total fat mass due to reduced large adipocyte. Alteration in cellularity also includes reduced ATM content, along with transformation from M1 to M2 polarization state. The last confirmed observation is dramatically modified adipokine secretion.

It was initially assumed that inclusion of n-3 PUFA is associated with remarkable changes in the plasma levels of two key adipokines, adiponectin and leptin. Further studies demonstrate that the induction of adiponectin by n-3 PUFA is adiposity-independent, since reduction of fat content due to caloric restriction hardly regulates adiponectin levels [90, 97, 98]. Based on emerging data highlighting the negative regulation of n-3 PUFA on leptin [99], conflicting results demonstrate that, in contrast to adiponectin, plasma leptin levels decrease with caloric restriction, an effect depending on body fat mass [100]. We cannot exclude the possibility that alteration in leptin is secondary to inhibition of enlarged AT caused by n-3 PUFA, rather than a direct consequence of n-3 PUFA manipulation. Other adipokines visfatin and apelin are also regulated corresponding to n-3 PUFA treatment without alteration in circulating levels, suggesting that both of them are incompetent to mediate systemic influence [101]. With regard to proinflammatory mediators derived from AT, dietary interventions with n-3 PUFA have been found to reduce TNF-*α*, IL-6 levels in AT [102, 103]. Given depot-specificity, n-3 PUFA reduces the expression of inflammatory genes of both gonadal and subcutaneous fat depots, suggesting the prevention against AT inflammatory may be apply to different fat depots [90]. The mechanism concerning adipocyte biological functions unveils that n-3 PUFA efficiently incorporates into AT, making their way into the membrane phospholipids and TAG lipid droplets, which is regarded as a mechanism for its preferential effects in AT [104–107]. Overall, these observations indicate that n-3 PUFA brings about a shift from a proinflammatory microenvironment to one of reduced inflammations (Figure 2).

### 5.3. Systemic Benefits

As mentioned, AT is responsible for the major form of crosstalk between insulin-responsive tissues. Thus, any treatment aimed at normalizing AT storage and secretory functions will alleviate the inflammatory state and lead to a global improvement in insulin sensitivity. Admittedly, the anti-inflammatory benefits of dietary n-3 PUFA involve modification in different tissues (liver, skeletal muscle, and AT) [108], which is not the interest of present review. Instead, this part is focused on systemic benefits of n-3 PUFA mediated by AT.

A large body of evidences demonstrates that n-3 PUFA reduces lipolysis in AT, resulting in reduced NEFA release into circulation [52, 108]. The modulation of lipoprotein lipase, hormone-sensitive lipase and fatty acid synthase is proposed as a possible mechanism in which n-3 PUFA inhibits lipolysis in AT [109].

In parallel, beneficial effect of n-3 PUFA on systemic glucose and lipid homeostasis heavily relies on circulating levels of adiponectin that is recognized as a critical mediator in improving insulin sensitivity. Given its insulin-sensitizing effect, it makes sense that improved insulin action observed with n-3 PUFA treatment in adiposity, liver, and skeletal muscle is mediated by adiponectin. Of note, this adipocyte-derived hormone directly increases glucose utilization and fatty acid oxidation in liver and skeletal muscle via stimulating the activation of 5′-AMP-activated protein kinase (AMPK) [110] and PPAR*γ* [111], which is considered as the answer to this insulin-sensitizing effect of adiponectin. Actually, recent molecular studies illuminate the signal transduction of adiponectin in muscle cells [112], warranting that stimulation of adiponectin secretion induces mitochondrial biogenesis.

Recent results have increased interest in the role of hypothalamus inflammation playing in the pathogenesis of obesity. As an early and determining factor in the installation and progression of obesity, hypothalamic inflammation is a cardinal mechanism leading to the anomalous control of energy intake and expenditure [113, 114]. The substitution of saturated fat by n-3 PUFA in the diet reduces diet-induced hypothalamic inflammation and corrects the response to nutrient sensing signals [63].

N-3 PUFA minimizes the insulin resistance in high fat-fed mice in an adiponectin-dependent manner [100]. Additionally, insulin and nonesterified fatty acid are also affected predominantly by n-3 PUFA administration [100]. Taken together, the beneficial effects of n-3 PUFA on metabolic derangements are mediated in part by alleviation of AT inflammation, especially resulting from decreased secretion of proinflammatory adipokines, increased secretion of adiponectin, and induced synthesis of proresolving lipid mediators.

## 6. Intracellular Regulatory Mechanism Affected by n-3 PUFA

**Figure 3:**
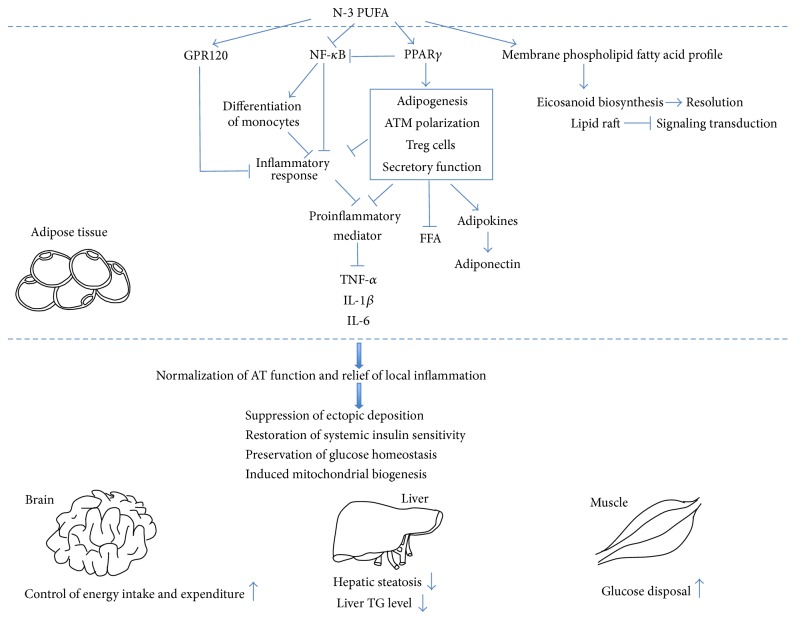
Putative mechanism by which adipose tissue mediates the systemic benefits derived from n-3 PUFA consumption. N-3 PUFA reduces adipose tissue inflammation via changing membrane phospholipid composition and activity of signaling pathways. The manipulation of membrane phospholipids composition and lipid raft microdomain with n-3 PUFA modify downstream processes mediated by membrane, such as signaling transduction, gene expression, and eicosanoid biosynthesis. Increased biosynthesis of resolvin and protectin contributes to resolution process. PPAR*γ* activation mediated by n-3 PUFA leads to increased number of small adipocytes, M2 macrophages, and Treg cells, while normalizing secretory function featured by induced adiponectin synthesis, inhibited lipolysis, and reduced pro-inflammatory mediator secretion. As anticipated, NF-*κ*B inhibition, which is partly dependent on PPAR*γ* activation, downregulates the expression of proinflammatory gene directly and indirectly via determining the differentiation of monocytes. GPR120 is the functional receptor making response to n-3 PUFA and mediates the anti-inflammatory benefits by repressing several inflammatory signalings. The improved storage and secretory functions of adipose tissue lead to relief of AT-specific inflammation, followed by global improvement of metabolic profile. Adipose-specific blunting of inflammation primarily favors the functional improvement of several organ systems involving liver, skeletal muscle, and brain.

The previous section summarizes the broad range of anti-inflammatory actions exerted by n-3 PUFA. Various mechanisms are directly or indirectly involved in explaining the influence of n-3 PUFA on metabolic events, including, but not limited to, the changes in membrane composition and intracellular metabolite levels concerning signaling pathways (Figure 3). Here, four approaches which are identified as the molecular targets of the anti-inflammatory effect of n-3 PUFA are described in detail, since the transcription of inflammatory genes determines the production of inflammatory cytokines by certain cells, which plays a direct as well as central role in inflammatory cascade. Activity of transcription factors mastering the inflammatory signaling will potentially modulate inflammation. Moreover, the integration of important cell-signaling pathways engaged in n-3 PUFA-mediated anti-inflammatory functions has only begun to be investigated and has not yet been clearly defined. A former review summarizes that the major role for PPARs is in the trans-suppression of inflammatory gene activation by negatively interfering with the nuclear factor *κ*B (NF-*κ*B) [115]. In C2C12 myotubes, we have discovered that the suppressive effect of EPA on NF-*κ*B activation is mediated via PPAR*γ* activation [116]. Similarly, the expression of PPAR*γ* in LPS-stimulated macrophages augments the inhibitory effect of inhibitor on NF-*κ*B activity [117]. As a complement to above observation, Zúñiga and colleagues [118] figure out an antagonistic effect of PPAR*α* on NF-*κ*B-controlled transcription of pro-inflammatory mediators.

### 6.1. Membrane Phospholipid Fatty Acid Profile

PUFA is a key structural and functional component of the phospholipids in cell membranes and the most common PUFA in the membrane phospholipids of macrophages, neutrophils, and lymphocytes is the n-6 PUFA AA [53]. In contrast to high proportion of AA, much less EPA and DHA are found in the cell membrane phospholipids. However, n-3 PUFA supplement in the diet of animals or healthy human demonstrates a significant incorporation of EPA and DHA into total AT lipids, liver TAG and phospholipid fractions, and brain phospholipids [119]. Corresponding to n-3 PUFA supplementation, mice exhibit the accumulation of n-3 PUFA in membrane phospholipids of immune cells coupled with reduced AA content [77]. The increased proportion of n-3 PUFA in the membrane phospholipids likely contributes to its anti-inflammatory effect of inhibiting production of proinflammatory mediators secreted by mononuclear cells [85]. As mentioned before, similar incorporation is observed in adipocytes. Researches attempting to illuminate the influence of dietary n-3 PUFA on the lipid composition and metabolism of adipocytes disclose that rats fed the n-3 PUFA have significantly lower concentrations of serum triglycerides, cholesterol, and insulin, concomitant with higher unsaturated to saturated fatty acid ratio observed in adipocyte membrane phospholipids [105]. Conceivably, the improvement of insulin sensitivity is also positively correlated with higher incorporation of n-3 PUFA in adipocyte membrane phospholipids [104].

It is thought that n-3 PUFA displaces AA from plasma membranes, decreasing its availability as a precursor of inflammation associated mediators. Inhibitory effect of n-3 PUFA on the release of substrate AA also is attributed to the suppressed phospholipase activity [76]. Irrespective of substrate accessibility, alteration of metabolites results from decreased AA metabolism due to the inhibition of cyclooxygenase (COX) activity [120]. This seems the possible explanation for the mechanism in which n-3 PUFA affects lipid-mediator profiles. The n-3 PUFA substitution of AA into adipocyte membrane phospholipids results in decreased level of prostaglandin E_2_ (PGE_2_) and subsequent downregulated fatty acid synthesis enzyme activity [69], which curbs adipocyte hypertrophic. On the other hand, decreased formation of prostaglandin D2 (PGD2) and its derivatives, known as PPAR*γ* ligands, presumably explains the effects of n-3 PUFA on proliferation and maturation of adipocytes [44]. Interestingly, insulin action is positively correlated with the fatty acid unsaturation index in membrane [104], whereas related mechanism remains obscure.

A second aspect of the alternation in cell membrane phospholipid fatty acid involves lipid raft in which key signal transduction proteins are localized, such as the tyrosine kinase lck and the signaling molecule linker of activated T cells [121]. Exposed to n-3 PUFA treatment, Jurkat T cell ends in marked enrichment of n-3 PUFA in lipids from isolated raft, with selective displacement of signaling proteins from raft in PUFA-treated T cells due to altered raft lipid composition [122]. Later data collectively demonstrate that modified raft lipid environment affects the membrane subdomain distribution of proteins involved in the interleukin-2 receptor (IL-2R) and toll-like receptor (TLR-4) signaling pathway [123, 124]. For now, disrupting rafts with n-3 PUFA and subsequent impact on immune cell function are centered on T cells, macrophages, and B cells, considered as the answer to immunosuppression property of n-3 PUFA.

The manipulation of membrane phospholipids composition and lipid raft microdomain with n-3 PUFA modify downstream processes mediated by membrane, such as signaling transduction, gene expression, and eicosanoid biosynthesis.

### 6.2. Peroxisome Proliferator-Activator Receptors (PPARs)

As lipid-activated transcription factors, PPARs contribute to attenuating inflammatory response. Numerous agonists of the related receptor PPAR have anti-inflammatory activity [125, 126]. Thiazolidinedione- (TZD-) induced activation of PPAR*γ* is reported to relieve hyperglycemia, hyperinsulinemia in vivo, reducing secretion of FFA, TNF-*α*, and leptin concomitantly [96]. PPAR*γ*, originally described in differentiating adipocytes, severs as a master regulator of adipogenesis and the target for the insulin-sensitizing drugs [127, 128]. Admittedly, forced expression of PPAR*γ* in the fibroblasts makes them differentiate into adipocytes [129] and activation of PPAR*γ* indeed contributes to increasing population of small adipocytes, but the total mass or the total triglyceride content of WAT remains unchanged with the number of large adipocytes decreasing [96]. Compared with large mature adipocytes, small immature ones seem less competent in secreting proinflammatory cytokines [130]. This shift toward smaller adipocytes seems to be partially responsible for the favorable metabolic effects of PPAR*γ* activators. With regard to the secretion of adipokines, PPAR*γ* agonists have been reported to increase circulating levels of adiponectin [131]. Moreover, PPAR*γ* null mice and in vitro experiments all demonstrate that the increased synthesis and secretion of adipokines by n-3 PUFA are dependent on PPAR*γ* [132, 133].

The expression of PPAR*γ* is also observed in cells of immune system and central nervous system. PPAR*γ* activation stimulated by short-term glitazones treatment increases infiltration of M2 in AT [134]. Macrophage-specific disruption of PPAR*γ* impairs development of alternatively activated M2 phenotype and predisposes individuals to diet-induced obesity [135]. Providing that PPAR*γ* is indispensable in the development of M2 phenotype, PPAR*γ* inactivation indirectly increases production of proinflammatory cytokine [136]. Similarly, PPAR*γ* activation suppresses monocyte elaboration of proinflammatory cytokines, including IL-1*β*, TNF-*α*, and IL-6 [137]. Unexpectedly, inactivation of PPAR*γ* in macrophages has unimaginable broad effects on significant glucose intolerance and impaired whole-body insulin resistance in lean mice fed a normal diet [136]. VAT-resident Foxp3^+^ Treg cells also specifically express PPAR*γ* which interacts with Foxp3 [138]. Foxp3-dependent PPAR*γ* conditional knockout mice demonstrate that Treg cell-specific deletion of PPAR*γ* leads to reduced number of Treg cells specifically in VAT, resulting in an increase in VAT infiltration of macrophages and monocytes. Additionally, stimulation with TZD drugs specifically increases VAT-resident Treg cells in the obese mice fed high-fat diet, with improving insulin sensitivity synchronically. These results collectively indicate that the therapeutic effect of TZD drugs partly is attributed to accumulation of Foxp3^+^ Treg cells in VAT. Naturally occurring Foxp3^+^ Treg cells produced by thymus possibly sense activator, as a result, PPAR*γ* expression is upregulated with migration of Foxp3^+^ Treg cells to the VAT. Alternatively, in parallel with accumulation of VAT-resident Foxp3^+^ Treg cells, augmentation of such suppressive activities of PPAR*γ*-expressing Treg cells achieved by ligand activation may enable better control of inflammation in obesity.

In conclusion, PPAR*γ* may act on differentiating adipocytes, macrophages, and Treg cells to alleviate AT inflammation and restore insulin sensitivity. The other mechanism involved in the effect of PPAR*γ* on anti-inflammation remains further investigation. High concentration of n-3 PUFA has been reported to be active PPAR*γ*, whereas their metabolites are stronger agonists [139]. Anti-inflammatory effects of n-3 PUFA are known to act at least in part through activation of PPAR*γ*. Although this speculation seems arguable, since n-6 PUFA, the stronger activator of PPAR*γ*, exhibits opposite effects. However, we are supposed to keep in mind that n-6 PUFA likely assumes their proinflammatory responsibility via other mechanism which is powerful enough to counteract the anti-inflammatory effect mediated by activation of PPAR*γ*.

Another candidate mechanism responsible for the anti-inflammatory action of n-3 PUFA is the removal of detrimental eicosanoids via *β*-oxidation, which is likely stimulated by PPAR activation [140].

### 6.3. Nuclear Factor *κ*B (NF-*κ*B)

Consistent with its central role in inflammatory signaling pathways, NF-*κ*B is widely investigated in the linkage between inflammatory and metabolic responses. Once activated by extracellular stimuli, NF-*κ*B promotes inflammation through initiating expression of genes encoding for inflammatory-related proteins in a very wide range of cell types, including macrophages, hepatocytes, and adipocytes. Additionally, NF-*κ*B directs the differentiation of distinct immune cell types by regulating expression of inflammatory mediators. NF-*κ*B-dependent differentiation of monocytes into either M1 or M2 macrophages in response to cytokines produced by immune cells also accounts for the involvement of NF-*κ*B in inflammation associated metabolic disorders, taking distinctive functions of M1 and M2 macrophages into consideration. Furthermore, activated NF-*κ*B promotes macrophage relocalization and activation, and more proinflammatory cytokines secretion augments macrophage activation and recruitment to the inflamed site [141]. Unexpectedly, different from a local and modest inflammatory response caused by IKK-*β* induced NF-*κ*B activation in hepatocytes [142], inactivation due to IKK-*β* deficiency in myeloid cells favors global insulin sensitivity [143]. Additionally, inactivation of NF-*κ*B by IKK-*β* deletion significantly diminishes the expression of inflammatory mediators [144]. Not only in peripheral metabolic tissues, recent study even discloses that in hypothalamus, NF-*κ*B which functions as the pro-inflammatory master switch is blamed for dysregulation of energy balance after sensing metabolic signals produced by overnutrition [113]. Brightly discriminated from the inflammatory reactions featured by chaotic release and powerful action of many inflammatory cytokines in nonneuronal cells, forced activation of hypothalamic IKK-*β*/NF-*κ*B interrupts central insulin/leptin signaling and actions in a neuron-specific and noncytokine way, resulting in increased intake of high-fat food and weight gain. By contrast, suppression of IKK-*β* either broadly or locally significantly protects against obesity and glucose intolerance [113], suggesting that inflammatory response in the peripheral metabolic loci trigged by overnutrition no longer causes functional defect in tissue-specific manner. Instead, metabolic inflammation and related inflammatory mediator are connected to the dysfunctions in the central nervous system.

In the quiescent state, NF-*κ*B remains inactive in the cytoplasm binding to the inhibitory protein I*κ*B. Phosphorylation of this inhibitory subunit releases NF-*κ*B, followed by NF-*κ*B translocation into nucleus where it controls the transcription of its target genes. Consistent with other researches based on THP-1 cells [87], we have found that n-3 PUFA inhibits NF-*κ*B activity in myotubes in vitro by preventing the degradation of I*κ*B*α* [145]. The explanation for the regulation of cytokines production caused by n-3 PUFA is the changes in inflammatory gene expression, which is partly ascribed to NF-*κ*B suppression with decreased I*κ*B phosphorylation [86, 87]. Notably, NF-*κ*B is enhanced or unaffected with saturated fatty acids and n-6 PUFA treatments [86, 146], validating that the inactivation of NF-*κ*B is exclusive to n-3 PUFA instead of a general lipid effect. Based on accessible reports, inhibitory effects of n-3 PUFA on NF-*κ*B activation lead to related gene expression, including IL-1, IL-1*β*, and TNF-*α*.

### 6.4. G Protein-Coupled Receptor 120

It has been reported previously that G protein-coupled receptor 120 (GPR120) functions as a signaling molecule for a wide array of cellular functions in response to unsaturated long-chain FAs [147]. Tissue expression pattern further indicates that GPR120 is the only lipid sensing GPR highly expressed in specialized proinflammatory tissue and cells [59], suggesting its critical role in development of obesity. Subsequent researches using human genetics approaches identify loss-of-function GPR120 gene variants that caused increased incidence of obesity and related sequelae, especially in the context of high-fat diet [148]. Interestingly, dysfunctional receptor underscores the inefficiency to transduce the signal of long-chain fatty acid [148], opening novel avenues of researches for antiobesity effects associated with n-3 PUFA. Actually, it has been already confirmed that GPR120 is the functional receptor making response to n-3 PUFA and mediates the anti-inflammatory benefits by repressing TRL2/3/4 and TNF-*α* inflammatory signaling in a *β*-arrestin2 dependent way, coupled with insulin sensitizing actions [59]. In more detail, following n-3 PUFA-stimulated internalization of the GPR120/*β*-arrestin2 complex, *β*-arrestin2 associates with TAK1 binding protein 1 (TAB1), blocking the association of TAB1 with activated kinase 1 (TAK1), which dampens downstream signaling to the IKK*β*/NF-*κ*B and JNK/AP1 system [59]. A similar receptor dependency for the anti-inflammatory effects of n-3 PUFA is observed in hypothalamus and Kupffer cells [63, 91].

## 7. Conclusion

Prolonged nutrient excess elicits infiltration of macrophages and other immune cells in AT, resulting in uncontrolled secretion of proinflammatory cytokines and a state of chronic low-grade inflammation. In turn, these released cytokines affect other organs, such as skeletal muscle, liver, and brain, ultimately ending up with metabolic abnormalities systemically. Undoubtedly, AT is the epicenter of the obesity, since inflamed AT is the primary source blamed for proinflammatory cytokines. The overall impacts of n-3 PUFA on AT biology and metabolism fall into three categories: storage, secretory function, and inflammation. Decreased storage and normalized secretion finally achieve adipose-specific blunting of inflammation. Aligning with the emerging view, AT lies at the crossroad of nutrient sensing, metabolism, inflammation, and endocrine. As anticipated, results from clinical and molecular studies have converged to highlight the broad spectrum of protective effects of n-3 PUFA in obesity and comorbidity. There is considerable crosstalk between AT and a wide array of organs, underscoring the difficulty in dissecting tissue-specific effects of n-3 PUFA and additional benefits mediated by AT. Recapitulating the phenotypical and metabolic date, we easily come to the conclusion that specific treatment results in a certain phenotype. However, elevated circulating adiponectin derived from AT due to dietary n-3 PUFA is an easily ignored contributor to restoration of insulin signaling in liver and muscle. Identification of the “missing link” between treatment and direct executor is an important next step towards our understanding of the actions of n-3 PUFA.

More recently, nutrients and environmental factors have been shown to induce epigenetic modifications [149], which is definitely a most rapidly expanding field in biology. Mapping of epigenetic marks, such as DNA methylation, histone modifications, and nucleosome positioning, is critical for understanding the regulatory mechanism for gene expression, chromatin remodeling factors, and noncoding RNA expression influenced by diet constitutes. Since the pathophysiology of obesity is concomitant with extensive gene expression changes, special emphasis is put on identification epigenetic changes induced by obesity and mechanism through which epigenetics contribute to obesity [150]. It would appear that future studies might be needed to establish and validate the relationship between dietary n-3 PUFA and antiobesity effect and how epigenetic mechanism may link them. Although the effect of dietary n-3 PUFA on the increased expression of leptin seems unrelated to promoter CpG methylation, given that feeding mice with n-3 PUFA diet is unable to affect CpG methylation in the leptin gene promoters [151]. The reasonable explanation may be the irrelevance between n-3 PUFA and increased leptin, which has been suggested before. Otherwise, we cannot rule out the possibility that histone modification, rather than DNA methylation, is the determinant elucidating the regulatory influence of n-3 PUFA on leptin expression in obesity.

The fact that not all AT expansion is necessarily associated with pathological changes makes us rethink our understanding towards obesity. Despite having excessive fat accumulation, a unique subset of obese individuals seem metabolically healthy, bypassing all of the aforementioned pathological phenotype associated with obesity [152]. It is supposed to reconsider and revalue obesity professionally in terms of metabolic disturbances and metabolic parameters, rather than arbitrary judgment based on fat mass. Accordingly, n-3 PUFA is never a panacea for dramatic loss of AT mass compared with energy restriction, but it is proficient in protecting from obesity-induced metabolic abnormalities, regardless of weight loss. In this regard, n-3 PUFA is a qualified nutritional regimen fighting against metabolic disorders. The anti-inflammatory and immune-regulatory effects of n-3 PUFA would be the hot issue worthy of further investigation rather than having antiobesity effects as the endpoint.
